# Giant desmoid tumor of the abdominal wall: a case report

**DOI:** 10.1016/j.ijscr.2025.111304

**Published:** 2025-04-15

**Authors:** Mohamed Amine Tormane, Ghazi Laamiri, Ichraf Jbir, Nada Ltifi, Mahdi Bouassida, Hassen Touinsi

**Affiliations:** Department of General Surgery, Hospital Mohamed Taher Maamouri, Nabeul, Tunisia; University Tunis El Manar, Faculty of Medicine of Tunis, Tunisia

**Keywords:** Abdominal wall, Benign, Case report, Desmoid tumor, Fibroblastic

## Abstract

**Introduction and importance:**

Desmoid tumors are a rare type of benign fibromatosis. Patients often present with a painless abdominal mass. Here, we report a case of a giant desmoid tumor of the abdominal wall that was successfully treated with wide local excision.

**Case presentation:**

We present the case of a 48-year-old female who developed an abdominal mass over the past year. Imaging revealed a well-circumscribed mass on the left side of the abdomen, which was treated with wide local excision. Immunohistochemical analysis of the specimen confirmed the diagnosis of a desmoid tumor. The patient had an uneventful postoperative recovery and remained in remission during an 18-month follow-up period.

**Discussion:**

Desmoid tumors are very rare benign lesions that typically affect young women. Abdominal wall localization is more common in individuals with Gardner syndrome. Surgery remains the primary treatment, with the approach depending on the size and location of the lesion. Radiotherapy is considered an alternative for tumors that are unresectable or incompletely excised. The definitive diagnosis requires immunohistochemical analysis of the specimen.

**Conclusion:**

Desmoid tumors are benign fibromatoses that can occur in the abdominal wall.

Symptoms are typically non-specific. Surgery is the main treatment, always aiming for radical resection with free margins.

Histological analysis of the surgical specimen is crucial for confirming the diagnosis.

## Highlights

• Abdominal wall desmoid tumors are non-cancerous fibromatoses.
• Symptoms are typically non-specific.
• Surgical resection is the only radical treatment.
• Radiotherapy is considered an option for unresectable or incompletely excised tumors.
• Immunohistochemical analysis is crucial for a definitive diagnosis.

## 1 Introduction and importance

Desmoid tumors, also known as aggressive fibromatosis, are a rare type of benign monoclonal tumor arising from connective tissue, particularly fibroblasts [1]. They account for 0.03 % of all neoplasms and 3 % of soft tissue tumors, with an estimated incidence of 2 to 4 cases per million people [2].

These tumors typically occur between the ages of 25 and 40, with a higher prevalence in women of childbearing age [3]. They are most commonly found in the upper arms, abdominal wall, and mesentery, and are classified into three types: Abdominal wall, intra-abdominal, and extra-abdominal [4]. The disease is locally aggressive and has a very high recurrence rate, reaching 56 %. Surgical excision is regarded as the standard treatment [5].

Herein, we present a rare case of a 48-year-old woman with a giant desmoid tumor of the abdominal wall, successfully treated with wide local excision.

This work has been reported following the SCARE 2020 criteria [6].

## 2 Presentation of a case

**Fig. 1:**
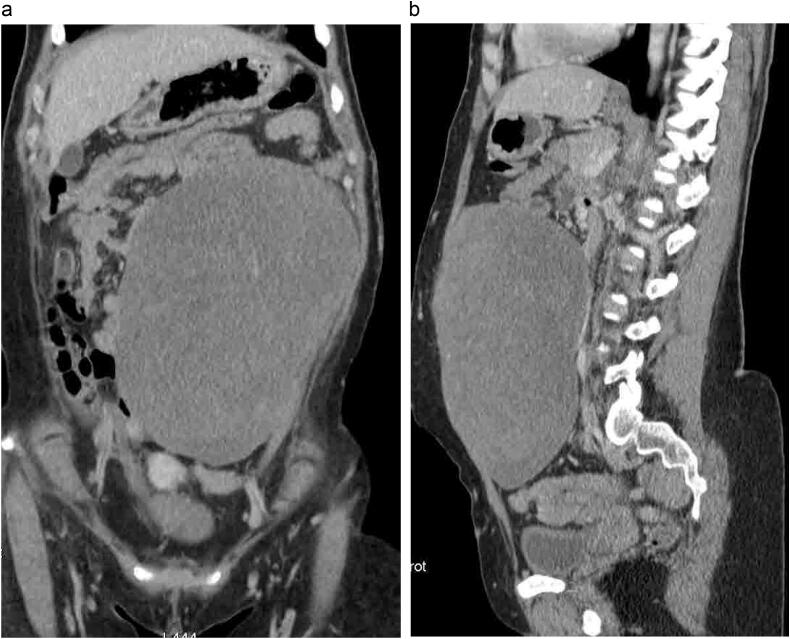
a: The coronal CT scan section shows a well-circumscribed mass on the left side of the abdomen b: The sagittal CT slice reveals the extent of the lesion.

A 48-year-old female presented with a painless left anterior abdominal mass that had been gradually increasing in size over the past year. She reported no history of trauma, abdominal surgery, or intestinal polyposis. She was a non-smoker, did not consume alcohol, and had no significant medical or family history. On physical examination a firm, non-tender and immobile mass was palpated in the left lower quadrant of the abdomen, extending from the region of the rectus abdominis muscle towards the iliac crest measuring approximately 18 cm. The overlying skin showed no signs of erythema or ulceration. Blood tests, including tumor markers, were within normal range. An abdominal CT Scan revealed a bulky, well-circumscribed mass measuring 18 × 14 × 11 cm, with a density similar to that of muscle. The lesion was localized to the rectus abdominis muscle on the left side extended to the iliac crest, with no evidence of invasion into intra-abdominal organs or distant structures (Fig. 1a, b).

**Fig. 2:**
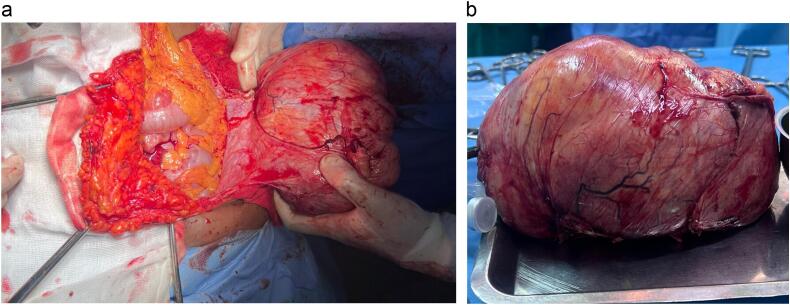
a,b: The specimen.

During laparotomy, a multilobulated mass was discovered, involving various layers of the left abdominal wall, without evidence of extension or secondary intra-abdominal involvement (Fig. 2a).

**Fig. 3:**
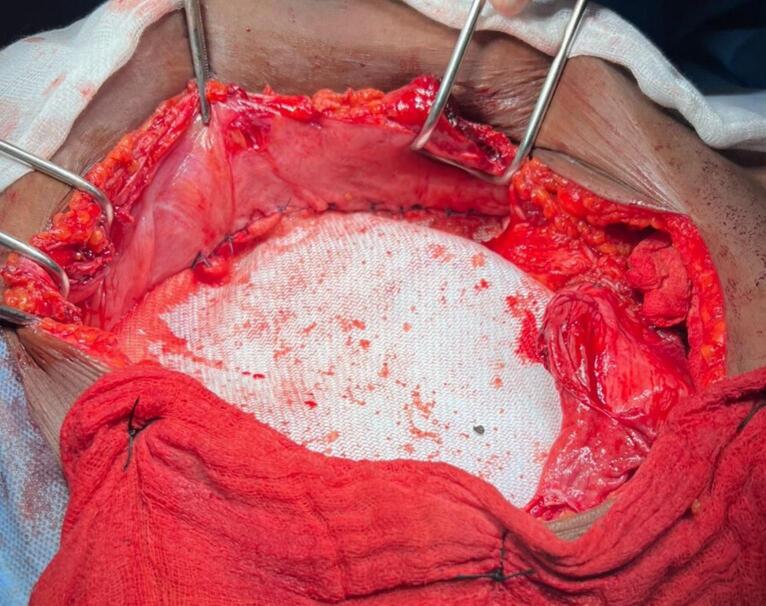
Intraoperative view showing the repair of the defect using a polypropylene mesh.

A radical resection of the mass was performed, including removal of the subcutaneous tissue, affected muscle, adjacent fascia, and peritoneal layer, ensuring a 1 cm peripheral margin of healthy tissue (Fig. 2b). The repair required detailed pre-operative assessment to determine the defect size and appropriate reconstruction strategy. A polypropylene mesh was selected for its durability and tissue integration properties. During the repair, the mesh was meticulously anchored to the healthy fascia and musculature with an optimal degree of tension to preserve mechanical integrity and accommodate dynamic movements of the abdominal wall. To minimize postoperative complications, the surgical field was extensively irrigated with antiseptic solutions, and the mesh was positioned to enhance biocompatibility and long-term integration (Fig. 3). The resected specimen was sent for histological analysis. The patient had an uneventful postoperative course and she was discharged on the sixth postoperative day.

**Fig. 4:**
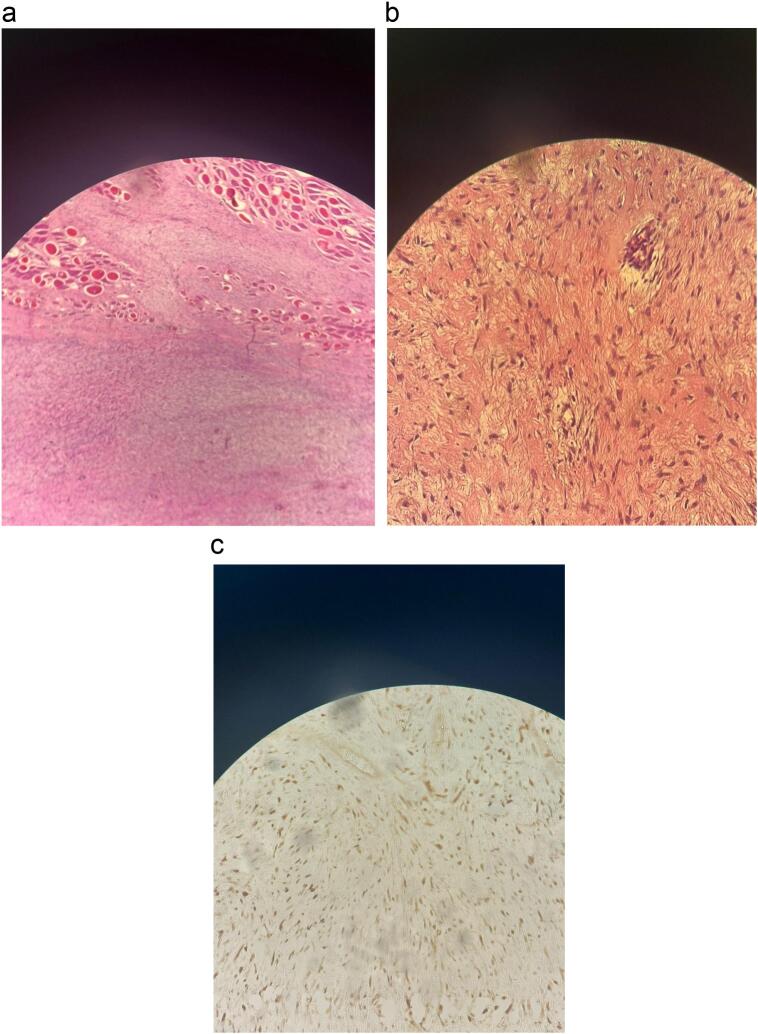
a: Microscopic examination shows infiltration of adjacent striated muscle by the tumor b: Microscopic examination reveals tumor cells with pale eosinophilic cytoplasm and finely structured chromatin, embedded within a collagen matrix. c: Immunohistochemical analysis showed positivity for β-catenin.

Macroscopically, the tumor had a firm, granular texture. The cut surface appeared pale and coarsely trabeculated, with no definitive capsule. Microscopic examination highlighted spindle-cell tumors infiltrating muscle tissues, including partially intact muscle fibers surrounded by spindle-shaped elements (Fig. 4a). The tumor cells demonstrated pale eosinophilic cytoplasm and chromatin structures, embedded in a collagen matrix interspersed with fibrotic areas (Fig. 4b). The surgical margins were clear. Immunohistochemical analysis showed positivity for β-catenin (Fig. 4c), while CD34 was negative. Based on these morphological and immunohistochemical features, a diagnosis of desmoid tumor without malignant features was confirmed. The patient remained in remission with good overall performance and no evidence of tumor recurrence or incisional hernia development after 18 months of follow-up. FAP was ruled out based on the absence of clinical features suggestive of this condition, including a lack of personal or family history of colorectal polyps, colorectal cancer, or other related extracolonic manifestations.

## 3 Clinical discussion

We report a successful radical resection of a giant desmoid tumor of the abdominal wall. The main strength of our work is the complete excision with intact margins, while the primary weakness lies in the late diagnosis and management of the disease.

Desmoid tumors are non-cancerous deep fibromatoses that develop from the fascia and muscle aponeurosis, particularly in the rectus and internal oblique muscles, along with their fascial layers. They may sometimes extend across the midline [7]. Approximately 3.7 new cases arise annually per one million people, primarily presenting as an extracolonic manifestation of familial adenomatous polyposis(FAP) [8].

They can be classified into five subgroups: Intra-abdominal, extra-abdominal, multiple, multiple familial, and those associated with Gardner's syndrome [3].

Abdominal desmoid tumors, which can develop in the abdominal wall, mesentery, or retroperitoneum, are more commonly seen in individuals with Gardner syndrome [3].

These tumors are usually associated with familial adenomatous polyposis (FAP), female gender, and occasionally with surgical trauma [9]. They typically affect young women, particularly during or after pregnancy. It has been demonstrated that fibroblasts exhibit a proliferative response to estrogen [10]. However, in our case, the patient is post-menopausal with no history of abdominal surgery and no personal or family history of colorectal polyps or other extracolonic manifestations.

Abdominal desmoid tumor generally presents as a mass measuring 5 to 15 cm, occasionally associated with weight loss and pain [11].

In our case, the patient presented with a painless left anterior abdominal mass.

CT scans are used to locate the tumor and detect any metastases. Desmoid tumors may appear homogeneous or heterogeneous and hypo-, iso-, or hyperintense compared with the attenuation of muscles. MRI shows hypointensity of the tumor on T1 and variable signal intensity on T2. Consequently, it is impossible to differentiate them from other solid tumors using morphological criteria [12].

Due to the lack of specific clinical or radiological features, the diagnosis of a desmoid tumor can only be definitively made through histopathological analysis of the excised specimen. Histology reveals extended fascicles of spindle cells with varying cell densities, showing few mitotic figures and lacking atypical nuclear divisions. There is widespread infiltration of cells into surrounding tissue structures. The cells are linearly arranged and separated from each other by collagen [11]. Muscle cell markers help distinguish desmoid tumors from fibrosarcoma and immunohistochemical response for actin can be partially positive [13]. In our case, immunohistochemistry analyses were positive for β-catenin.

Acute hematoma, lymphoma, rhabdomyosarcoma, liposarcoma, fibrosarcoma leiomyosarcoma, and primitive neuroectodermal tumor are among the differential diagnoses for rectus abdominis lesions [10].

The cornerstone of treatment for desmoid tumors remains resection with negative margins, especially for small and accessible forms [14]. Adjacent structures and intraperitoneal organs affected by the tumor should also be resected. Incomplete removal of the tumor or compromised excision margins can lead to local relapse [3].

The recurrence rate of abdominal wall desmoid tumors is 20–30 % and typically appears within six months of excision. No metastatic disease has been documented with this type of tumor [1,3,4,6].

No significant benefit has been observed with anti-inflammatory treatment, hormone therapy, or chemotherapy. The use of these therapies is limited to patients in whom resection is not technically feasible due to extensive tumor infiltration [15]. Radiotherapy is used for patients with unresectable or incompletely excised tumor. In fact, a comparative analysis showed significantly better control of local recurrence with radiotherapy combined surgical resection, compared with resection alone [16].

Based on the germline mutations and chromosomal aberrations of the APC alleles, Bright-Thomas et al. [17] Conducted a preclinical study using gene transfer to treat desmoid disease in familial adenomatous polyposis (FAP).

Further work in animal models of desmoid disease is required to assess the clinical impact of gene therapy, despite the success of transgene expression.

Recent advancements in the management of desmoid tumors have introduced promising therapeutic options. In 2023, the FDA approved Nirogacestat, a gamma-secretase inhibitor, for the treatment of progressing desmoid tumors, marking a significant milestone as the first systemic therapy approved for this condition. Nirogacestat targets gamma-secretase, a key enzyme involved in the growth of desmoid tumors, offering a novel mechanism of action [18]. Clinical trials, such as the DeFi study, demonstrated the efficacy of Nirogacestat in improving progression-free survival. In addition to reducing tumor growth, patients treated with Nirogacestat reported improvements in pain and quality of life [19]. This approval has transformed the treatment landscape for desmoid tumors, providing patients with a non-surgical option that addresses both tumor progression and quality of life concerns.

In summary, we present the case of a 48-year-old female with a giant abdominal wall mass suggestive of a desmoid tumor, suspected by CT scan, and successfully treated with surgical resection. Our case underscores the importance of radical resection with clear margins and immunohistochemical analysis of the excised specimen for definitive diagnosis and effective treatment.

## 4 Conclusion

Desmoid tumors are benign fibromatoses that can occur in the abdominal wall.

They are most commonly observed in female patients, particularly those with a history of abdominal surgery.

Symptoms are typically non-specific, with the tumor often presenting as a painless abdominal mass.

Surgery remains the primary treatment, always aiming for radical resection with free margins.

The definitive diagnosis is made through histological analysis of the excised specimen.

Non-surgical treatment, primarily radiotherapy, has shown variable and unpredictable outcomes and is considered an alternative for patients with inoperable lesions or as an adjuvant therapy.

## CRediT authorship contribution statement

Mohamed Amine Tormane and Ghazi Laamiri did the conception and design of the work, the data collection, and the data analysis and interpretation.

Ichraf Jbir and Nada Ltifi: did the critical revision of the article.

Mahdi Bouassida and Hassen Touinsi: did the final approval of the version to be published.

All authors read and approved the final manuscript.

## Patient consent

Written informed consent was obtained from the patient for publication of this case report and accompanying images. A copy of the written consent is available for review by the Editor-in-Chief of this journal on request.

## Ethical approval

Ethical approval for this study is not required. An exemption has been obtained by the ethics committee of the Mohamed Taher Maamouri hospital because the patient's anonymity was respected.

## Guarantor

Mohamed Amine Tormane

Ghazi Laamiri

## Provenance and peer review

Not commissioned, externally peer-reviewed.

## Sources of funding

This research did not receive any specific grant from funding agencies in the public, commercial, or not-for-profit sectors.

## Registration of research studies

None.

## Declaration of competing interest

No conflicts of interest.
